# Exercise-Induced Hyponatremia: An Assessment of the International Hydration Recommendations Followed During the Gran Trail De Peñalara and Vitoria-Gasteiz Ironman Competitions

**DOI:** 10.3389/fnut.2021.781229

**Published:** 2022-02-21

**Authors:** Diego López de Lara, Jorge Gabriel Ruiz-Sánchez, Martín Cuesta, Germán Seara, Alfonso Luis Calle-Pascual, Miguel Ángel Rubio Herrera, Isabelle Runkle, Joseph George Verbalis

**Affiliations:** ^1^Endocrinología Pediátrica, Hospital Clínico San Carlos, Instituto de Investigación Sanitaria Biomédica San Carlos (IdISSC), Madrid, Spain; ^2^Endocrinología y Nutrición, Hospital Universitario Fundación Jiménez Díaz, Instituto de Investigación Sanitaria Biomédica San Carlos (IdISSC), Madrid, Spain; ^3^Hospital Clínico San Carlos, Instituto de Investigación Sanitaria Biomédica San Carlos (IdISSC), Madrid, Spain; ^4^Georgetown University Medical Center, Washington, DC, United States

**Keywords:** exercise, sodium disorders, hyponatremia, hypernatremia, copeptin

## Abstract

**Introduction:**

Hyponatremia often occurs during the practice of endurance sports. We evaluated the impact on hyponatremia of the hydration recommendations of the Third International Exercise-Associated Hyponatremia Consensus Development Conference 2015 (3IE-AHCD) during the 2017 *Gran Trail de Peñalara* marathon (GTP) and the *Vitoria Gasteiz Ironman* triathlon (VGI).

**Methods:**

Prospective study of GTP and VGI athletes participating in four information sessions in the months prior to the events, to explain that hydration should only be according to their level of thirst, per the recommendations of the 3IE-AHCD. Consenting event finishers were included in final analysis. Pre- and post-race anthropometric and biochemical parameters were compared.

**Results:**

Thirty-six GTP (33 male) and 94 VGI (88 male) finishers were evaluated. GTP race median fluid intake was 800 ml/h, with 900 ml/h in the VGI race. 83.3% GTPfin and 77.6% VGIfin remained eunatremic (blood sodium 135–145 mmol/L). Only 1/36 GTP and 1/94 VGI participant finished in hyponatremia, both with a sodium level of 134 mmol/L. Fourteen percent of GTP, and 21.2% of VGI participants finished in hypernatremia, with no increase in race completion times. No participating athlete required medical attention, except for musculoskeletal complaints. Pro-BNP and Copeptin levels rose significantly. Changes in copeptin levels did not correlate with changes in plasma osmolality, nor total body water content in impedance analysis.

**Conclusions:**

Recommending that athletes' fluid intake in endurance events be a function of their thirst almost entirely prevented development of hyponatremia, without induction of clinically significant hypernatremia, or a negative repercussion on race completion times.

## Introduction

Hyponatremia often occurs during physical exercise, particularly during the practice of endurance sports (1–4). One of the postulated causes is an excess consumption of fluids. In fact, athletes taking part in long-distance events are often worried that their fluid intake (FI) will be insufficient, inducing them to drink in excess (5). The problem can be exacerbated by a reduction in the urine water excretion due to exercise-induced non-osmotic secretion of the antidiuretic hormone, arginine vasopressin (AVP). Indeed, exercise-induced hyponatremia is one of the most important medical problems encountered during marathons, ultra-marathons and Ironman distance triathlons, since it can be lethal. In fact, incidences ranging from 15 to 30% have been reported, and sodium levels below 120 mmol/L found in ~1% of athletes (1, 2, 6).

The optimum level of hydration for athletes participating in ultra-endurance events is a subject of controversy (7). Prior to the 1980's, athletes were encouraged to avoid consuming liquids while competing, which led to many experiencing dehydration and hypernatremia (8, 9). Over the next 20 years, they were encouraged to do the opposite, with recommendations emphasizing negative consequences of dehydration (8–10). However, this served to increase the incidence of hyponatremia, especially in the United States (11–14). In fact, more recent studies highlight the problems associated with hyperhydration (3, 5, 7, 15). The death of a triathlete due to cerebral edema during the 2015 Frankfurt European Ironman Championship, and the description of other fatal cases of acute hyponatremia owed to excessive FI, has alerted organizers of ultra-distance events, and re-opened the debate over the risk of hyponatremia vs. inadequate hydration strategies (5, 7).

Fluid losses through sweat and urine are highly dynamic, and vary from one athlete to the next. Furthermore, the type of sport, and weather, as well as volemia, will influence an individual athlete's needs for fluid replacement. Simply indicating that athletes drink as much as possible, or recommending fixed ranges of fluid intake, do not take these factors into account.

The Third International Exercise-Associated Hyponatremia Consensus Development Conference 2015 (3IE-AHCD) recommends that athletes should drink according to thirst (5)—no more, no less. The 3IE-AHCD also recommends placing provisioning posts every 5 km. in foot races, and every 20 km. in cycling races. Drinking when thirsty would permit the principal physiological factors involved in regulating body water, thirst and AVP secretion, to remain in control of water homeostasis.

The present study assesses the effect of the hydration recommendations for athletes made by the 3IE-AHCD on the incidence of hyponatremia. The events chosen for this analysis were the *Gran Trail de Peñalara* (GTP) and the Vitoria-Gasteiz Ironman (VGI) competitions. These two events were chosen given their prestige, quality of organization, and difficulty, as well as their similarity to events studied in prior articles on sports hyponatremia. Athletes were provided with the 3IE-AHCD recommendations, and thus encouraged to hydrate only when thirsty.

## Materials and Methods

### Study Design and Subjects

**Figure 1:**
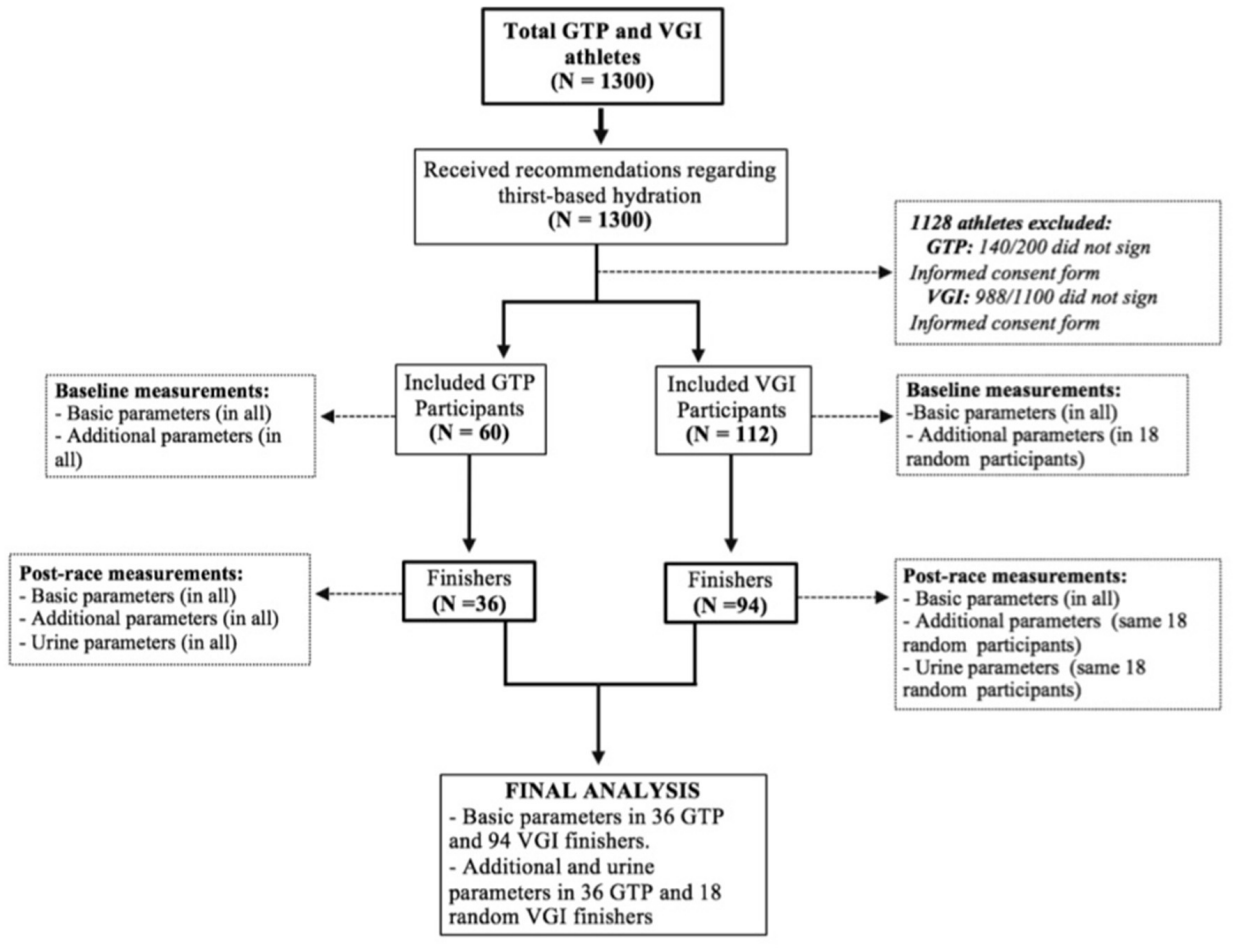
Algorithm of participant selection for the study. Basic parameters were as follows: medical history, demographic, fluid and salt intake, biometrics, impedance analysis, whole blood electrolytes and glucose, and calculated effective plasma osmolality. Additional parameters were as follows: measured plasma osmolality, serum copeptin, pro-BNP, urea, creatinine, leucocytes, Hemoglobin, hematocrit. Urine parameters were as follows: osmolality, sodium, and potassium.

Data for this prospective study were collected during the GTP and VGI competitions held on June 23rd and July 9th 2017, respectively. The study subjects were 18–65-year-old athletes/triathletes federated in Spain, recruited with the help of the Schools of Medicine and Physical Education of the *Universidad Complutense de Madrid*, the Innovation Unit of the San Carlos Health Research Institute, and the Pediatric and Endocrinology Departments of the *Hospital Cl*í*nico San Carlos (HCSC)*. Four information sessions were held with registered participants in the months prior to the events, to explain that they should only hydrate according to their level of thirst, as per the recommendations of the 3IE-AHCD. These recommendations were also e-mailed to all the subjects and posted on the event organizers' webpages and social networks (Supplementary Material). The day before each race, athletes were asked to agree to take part in the study. Only those signing the informed consent form and finishing their race were included in the final analysis. Figure 1 provides an overview of participants recruited and the analysis that was performed.

### The Gran Trail de Peñalara Race

In recent years, the popularity of mountain races covering very long distances and involving large changes in altitude—known as “trail” or “ultra-trail” events—has increased. These foot-races cover up to 166 km. and take place over mountain tracks. In 2010, the Real Sociedad Española de Alpinismo Peñalara inaugurated the GTP race in the Guadarrama sierra of Madrid. The race provides participants with points toward competing in the Mont Blanc Ultra-Trail race, considered the World Championship Ultra Trail event.

The GTP race is a circular, semi-self-sufficiency competition, covering 114 km, ranging from 1,315 to 2,428 m above sea level (m.o.s.l.), accumulated altitude gain: 4,474 m. Participants carry the minimum material guaranteeing their safety, with 30 h to complete the distance. Ten provisioning posts 5–10 km apart were set up along the route, providing water, Coca-Cola™ (1 mmol Na/100 mL), Aquarius™ (1 mmol Na/100 mL), Powerade™ (3.9 mmol Na/100 mL), salt tablets (200 mg NaCl), fruit, and energy bars. Maximum environmental temperature reached 33°C. Relative humidity was 61%. The event started at 6.30 AM. Sixty of the 200 athletes starting the race signed the Informed Consent Form.

### The Vitoria Gasteiz Ironman Race

This event, one of the most anticipated in Spain, is comparable to the most important European Ironman-length triathlons. Like other Ironman triathlons, it involves a 3,800 m swim, a 180 km cycling race, and a 42.2 km race on foot. Altitude ranges from 508 to 613 m.o.s.l. Provisioning stations were placed every 20 km of the cycling race and every 5 km of the race on foot, providing similar liquid, salt, and food resources as the GTP. Environmental temperature reached 31°C. Relative humidity was 57%. The event started at 7.30 AM. Of the 1,100 subjects starting the race, 112 signed the IFC.

### Data Collection

Demographic data and medical history were collected *via* questionnaire on the eve of the races. Biometric parameters and blood for biochemical measurements for analysis were collected 12–24 h prior to the races (“baseline”) in all consenting participants, and within 30 min of successful race completion. Urine samples were collected 15–90 min post-race. Post-race parameters included self-reported information on fluid and salt tablet intake. Pre- and post-race analysis was performed in all finishers, and included whole blood glucose, sodium, and potassium levels (VITROS 5600 Integrated System), as well as weight and height (stadiometer), and total body water (impedance analysis by an INBODY 270 device).

In all GTP finishers, and in a random sampling of 18 VGI finishers pre and post-race analysis of the following additional parameters was also performed: Hb, Hematocrit, leukocytes, serum urea and creatinine (Sysmex Xe-2100 analyzer) Plasma Osmolality (POsm) and Urine Osmolality (UOsm; Advanced Instruments 3300 osmometer), urine electrolytes (Beckman Coulter AU5800 analyzer), serum Copeptin (enzyme immunoassay using Cloud Clone reagents), and serum Pro-BNP (immunoluminescence using a Roche Elecsys E-170). Effective POsm (EPOsm) was calculated: 2xBNa^+^ + [blood glucose (mg/dL)/18]. Participants failing to complete their race were excluded (Figure 1).

Hyponatremia was defined as a total blood sodium level (BNa^+^) <135 mmol/L after correction for glycemia. Hypernatremia was defined as BNa^+^ >145 mmol/L, eunatremia as BNa^+^ 135–145 mmol/L.

### Statistical Analysis

Categorical variables were expressed as frequency rates, with group comparisons done by Chi-square tests for categorical variables. Continuous variables were described as mean (±standard deviation) when parametric, or median [interquartile range] if non-parametric, as indicated by the Kolmogorov-Smirnov or Shapiro-Wilk test. When a numeric variable was below the detection level of the assay, the lower limit of detection was used for statistical analysis. Comparative analysis of the quantitative variables was performed using Mann-Whitney *U* or Kruskal-Wallis tests when non-parametric, and Student's *T*-test or ANOVA tests when parametric. Correlation studies, with Pearson's or Spearman's method, were also performed, for parametric and non-parametric variables, respectively. Statistical significance was considered when *p* < 0.05 in two-tailed analysis. SPSS 25 (IBM Corp., Armonk, N.Y.) was used for analysis.

### Ethical Statement

All included subjects signed the ICF. Subject data were anonymized and protected in application of Spanish legislation. The study protocol was approved by the Ethics and Clinical Research Committee of the HCSC (internal code 17/018-E). Participants were not involved in the design, conduct, reporting, or dissemination plans of our research.

## Results

Thirty-six of the initial 60 enrolled GTP athletes completed the event, of which 33 (91.6%) were male. Mean finishing time was 18:20 h (range: 14:13–22:27 h). Ninety-four of the initial 112 consenting VGI athletes completed the race, of which 88 (93.6%) were male. All athletes that did not finish their race were excluded from analysis. Mean finishing time was 11:20 h (range: 8:92–13:48 h). No participant declared comorbidities or drug consumption. All race finishers reported drinking fluids without thirst before the race, and only when thirsty during the race. No athlete, regardless of whether they finished their race or not, was attended by the medical team for anything other than musculoskeletal complaints.

Table 1 displays parameters measured pre- and post-race in the entire group of finishers as classified by each race. Table 2 displays additional pre- and post-race biochemical data of all GTP and 18 VGI finishers, both according to race and grouped together.

**Table 1:** Pre-and post-race parameters in of finishers as classified by race.

	**Total**	**Gran Trail de Peñalara**	**Vitoria Gasteiz Ironman race**	***p* ^a^**
	**(*N* = 130)**	**(*N* = 36)**	**(*N* = 94)**	
Age, years	38 [33–43]	39 [31–44]	38 [33–43]	**0.779**
Sex male, *n* (%)	121 (93.1)	33 (91.7)	88 (93.6)	**0.695**
Height, cm	176.3 ± 7	175 [169–180]	178 [172–182]	0.025^*^
Weight (kg) pre-race	71.9 ± 8.4	70.6 ± 7.8	72.5 ± 8.7	**0.240**
Weight (kg) post-race	69.4 ± 8	68.3 ± 7.5	69.9 ± 8.3	**0.301**
***p* ^b^**	<0.001^*^	<0.001^*^	<0.001^*^	
BMI (kg/m^2^) pre-race	23.1 ± 1.7	23.3 ± 1.7	23 ± 1.8	**0.499**
BMI (kg/m^2^) post-race	22.3 ± 1.7	22.5 ± 1.7	22.2 ± 1.7	**0.379**
***p* ^b^**	<0.001^*^	<0.001^*^	<0.001^*^	
TBW (L) pre-race	46.3 [41.9–49.1]	45.1 [40.3–48.2]	46.8 [42.8–50]	0.030^*^
TBW (L) post-race	45.5 [41.2–48.3]	44.7 [40.2–47.8]	45.7 [41.7–48.8]	**0.147**
***p* ^b^**	<0.001^*^	**0.623**	<0.001^*^	
Finishing time (hh:min)	-	18:20 [14:13–22:27]	11:20 [8:52–13:48]	0.01^*^
Fluid intake (ml/h)	900 [700–1,100]	800 [625–1,000]	900 [700–1,100]	**0.639**
Fluid intake (ml/BMI/h)	39.2 [30.1–49.2]	36 [27.8–43.4]	40 [31.8–49.8]	**0.078**
Salt intake (g)	6.2 ± 5.9	9.1 ± 7.7	5 ± 4.7	<0.001^*^
**Whole blood parameters**				
Na^+^ (mmol/L) pre-race	139 [138–141]	140 [138–141]	139 [138–140]	0.037^*^
Na^+^ (mmol/L) post-race	143 [141–145]	142 [140–145]	143 [141–145]	**0.333**
***p* ^b^**	<0.001^*^	<0.001^*^	<0.001^*^	
Hyponatremia, n (%)	2 (1.5)	1 (2.8)	1 (1)	**1**
Hypernatremia, n (%)	25 (19.2)	5 (13.9)	20 (21.3)	**0.458**
K^+^ (mmol/L) pre-race	4.6 [4.2–5]	5.1 [4.6–5.4]	4.5 [4–4.8]	<0.001^*^
K^+^ (mmol/L) post-race	4.6 [4.3–5]	5 [4.4–5.2]	4.5 [4.3–4.9]	0.003^*^
***p* ^b^**	**0.614**	**0.052**	**0.089**	
Glucose (mg/dL) pre-race	84 [77–90]	85 [78–90]	84 [77–90]	**0.765**
Glucose (mg/dL) post-race	103 [88–118]	102 [89–121]	104 [86–117]	**0.617**
***p* ^b^**	<0.001^*^	<0.001^*^	<0.001^*^	
EPOsm (mOsm/kg) pre-race	283 [281–286]	284 [281–287]	283 [280–285]	**0.054**
EPOsm (mOsm/kg) post-race	291 [286–296]	291 [285–295]	292 [287–296]	**0.474**
***p* ^b^**	<0.001^*^	<0.001^*^	<0.001^*^	

a *Comparison between GTP and GIV.*

b *Comparison between pre- and post-race.*

* *p < 0.05.*

*Quantitative parameters are expressed in mean (± standard deviation) or median [interquartile range].*

*BMI, body mass index; TBW, total body water; Na^+^, sodium, K^+^, potassium; EPOsm, effective plasma osmolality*.

**Table 2:** Additional pre- and post-race parameters of athletes both grouped together and as classified by race.

	**Total**	**Gran Trail de Peñalara**	**Vitoria Gasteiz Ironman race**
	**(*N* = 54)**	**(*N* = 36)**	**(*N* = 18)**
**Additional blood parameters**			
POsm (mOsm/kg) pre-race	304 ± 4	304.3 ± 4.5	303.3 ± 4.2
POsm (mOsm/kg) post-race	316 ± 7	315.7 ± 7.3	316.4 ± 7.6
***p* ^b^**	<0.001^*^	<0.001^*^	<0.001^*^
Serum Copeptin pre-race (pmol/L)^a^	<15	<15	<15
Copeptin post-race (pmol/L)	88.5 [79.6–98.5]	86.5 [79.3–99.2]	90.6 [80.5–98.5]
***p* ^b^**	<0.001^*^	<0.001^*^	<0.001^*^
Serum Pro-BNP (pg/ml) pre-race	20 [18–30]	22 [18–31.8]	20 [20–22]
Pro-BNP (pg/ml) post-race	197 [117–414]	262 [112–519.3]	154 [118–221.3]
***p* ^b^**	<0.001^*^	<0.001^*^	<0.001^*^
Serum Urea (mg/dl) pre-race	41.9 ± 8.3	41.4 ± 7.8	42.8 ± 9.4
Urea (mg/dl) post-race	61.2 ± 13.8	65.3 ± 13.7	52.9 ± 9.7
***p* ^b^**	<0.001^*^	<0.001^*^	0.001^*^
Serum Creatinine (mg/dl) pre-race	0.9 [0.8–1]	0.9 [0.8–1]	0.9 [0.7–1]
Creatinine (mg/dl) post-race	1.1 [1.1–1.4]	1.1 [1.1–1.4]	1.2 [1.1–1.3]
***p* ^b^**	<0.001^*^	<0.001^*^	<0.001^*^
Leucocytes (x10^3^) pre-race	5.8 [5.1–7]	5.9 [5.3–7.5]	5.4 [4.9–6.1]
Leucocytes (x10^3^) post-race	14.7 [12.9–16.4]	14.1 [12.2–15.9]	16.1 [14.9–17.4]
***p* ^b^**	<0.001^*^	<0.001^*^	<0.001^*^
Hemoglobin (g/L) pre-race	15.1 [14.3–15.7]	14.8 [14.2–15.4]	15.7 [14.6–16.1]
Hemoglobin (g/L) post-race	15.2 [14.3–15.7]	15 [14.1–15.6]	15.3 [14.6–15.9]
***p* ^b^**	**0.718**	**0.619**	**0.154**
Hematocrit (%) pre-race	45.1 ± 3	44.4 ± 2.6	46.7 ± 2.9
Hematocrit (%) post-race	44.3 ± 3	43.8 ± 2.9	45.3 ± 2.6
***p* ^b^**	0.003^*^	**0.099**	0.003^*^
**Urine post-race parameters**			
Osmolality (mOsm/kg) post-race	908 [786–1033]	974 [855–1060]	822 [664–881]
Na^+^ (mmol/L)	68 [34–118]	66 [37–132]	68 [32–105]
K^+^ (mmol/L)	154 [128–210]	158 [129–225]	149 [124–156]

a *All copeptin levels were below 15 pmol/L. For statistical analysis, a value of 15 was used.*

b *Comparison of pre and post-race parameters.*

* *p < 0.05.*

*Quantitative parameters are expressed in mean (±standard deviation) or median [interquartile range].*

*POsm, plasma osmolality as measured by osmometer; Na^+^, sodium; K^+^, potassium*.

### Fluid Intake and Body Water Content

The median FI of the GTP and VGI athletes is shown in Table 1. In GTP, 19 of the 36 finishers (53%) drank 400–800 ml/h, as did 29 of the 94 finishing athletes (31%) in VGI. No athlete consumed <400 ml/h, nor >1,500 ml/h in either race. Analyzing both events together, we found a positive correlation between post-race TBW and FI in ml/h (*r* = 0.316, *p* < 0.001) and in ml/BMI/h (*r* = 0.175, *p* = 0.046).

GTP athletes presented a positive correlation between post-race BW and post-race TBW (*r* = 0.87, *p* < 0.001), and between the change in BW and that of TBW (*r* = 0.75, *p* < 0.001). Similarly, for VGI triathletes in the same variables: *r* = 0.91, *p* < 0.001; and *r* = 0.74, *p* < 0.001, respectively.

Four of the 36 (11.1%) GTPfin maintained their BW (i.e., less than a −1 to +1% change pre- to post-race), as did six of the 94 (6.3%) of VGIfin. No subject gained weight. Twelve (33%) of the 36 GTPfin lost between 1 and 3% of their BW, as did 31 of the 94 (33%) of VGIfin. Twenty of the 36 (56%) GTPfin lost >3% of BW, as did 57/94 (61%) of VGIfin. The maximum recorded loss was 5.2%. The highest EPOsm recorded in those with a loss >3% of BW was 305 mOsm/kg in GTP, and 307 mOsm/kg in VGIfin.

### Post-race Hyponatremia and Hypernatremia

**Figure 2:**
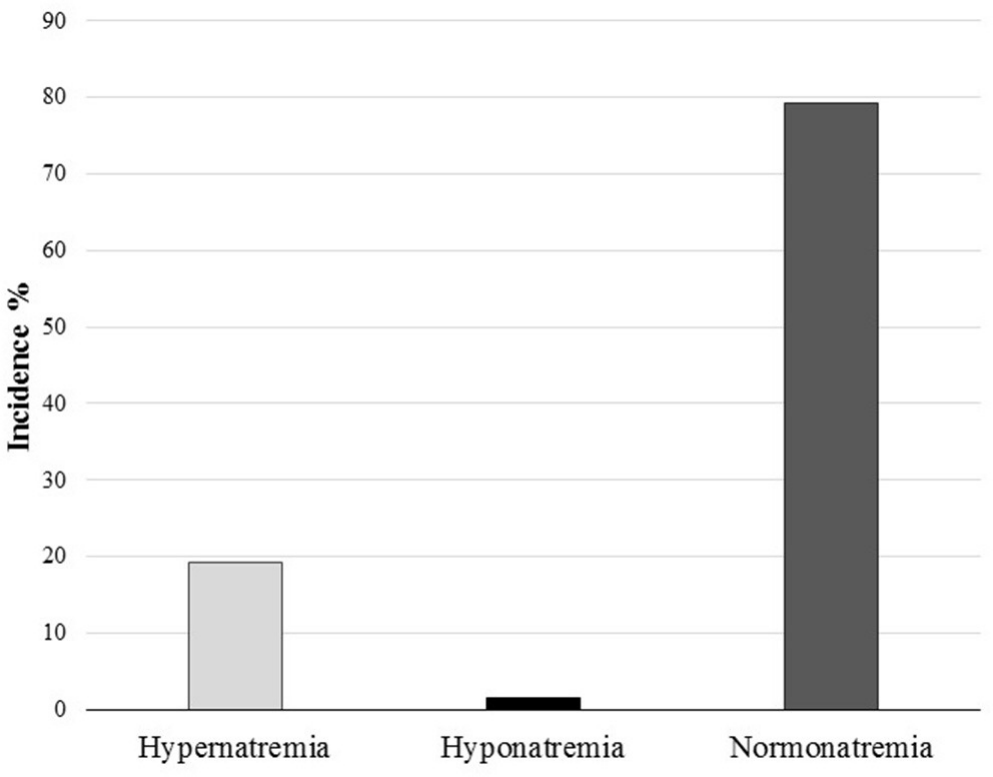
Total incidence of hyponatremia and hypernatremia during both races.

One hundred and three (79.2%) of the 130 finishers of either event remained eunatremic: GTP: 30/36 (83.3%), VGI: 73/94 (77.6%). Only 2 of the 130 (1.5%) athletes developed hyponatremia, GTP: 1/36 (2.8%), VGI: 1/94 (1%) (Figure 2). Characteristics of athletes developing hyponatremia are described in Table 3.

**Table 3:** Characteristics of the athletes who developed hyponatremia.

	**GTP athlete**	**VGI athlete**
Age, years	30	39
Sex	Female	Male
Finishing time (hh:min)	20:59	12:35
Fluid intake (ml/h)	600	700
Fluid intake (ml/BMI/h)	29	27.6
Salt intake (g)	7	5.6
**Impedance and biometric parameters**		
BMI (kg/m^2^) pre-race	20.7	25.4
Weight (kg) pre-race	55	73.3
Weight (kg) post-race	54.3	71.5
**Weight change (%)**	**−1.3**	**−2.5**
TBW (L) pre-race	29.9	46.2
TBW (L) post-race	30.6	46
**TBW change (%)**	**+2.34**	**−0.4**
BW% pre-race	54.3	63
BW% post-race	56.3	64.3
**BW% change (%)**	**+3.7**	**+2.1**
**Blood parameters**		
Na^+^ (mmol/L) pre-race	137	137
Na^+^ (mmol/L) post-race	134	134
K^+^ (mmol/L) pre-race	4.5	4.7
K^+^ (mmol/L) post-race	4.6	4.4
Glucose (mg/dl) pre-race	80	76
Glucose (mg/dl) post-race	125	102
EPOsm (mOsm/kg) pre-race	278	278
EPOsm (mOsm/kg) post-race	275	274
Copeptin (pmol/L) pre-race	<15	No data
Copeptin (pmol/L) post-race	145	No data
Pro-BNP (pg/ml) pre-race	18	No data
Pro-BNP (pg/ml) post-race	505	No data
Urea (mg/dl) pre-race	28	No data
Urea (mg/dl) post-race	65	No data
Creatinine (mg/dl) pre-race	0.7	No data
Creatinine (mg/dl) post-race	1.5	No data
**Urine parameters**		
Na^+^ (mmol/L)	20	No data
Osmolality (mOsm/kg)	511	No data
K^+^ (mmol/L)	118	No data

*BMI, body mass index; TBW, total body water; BW%, percentage of body water; Na^+^, sodium; K^+^, potassium; EPOsm, effective plasma osmolality*.

Hypernatremia was observed in 25 of the 130 (19.2%) athletes completing their race, as follows: GTP: 5 of the 36 (14%), VGI: 20 of the 94 (21.2%). No subject presented a BNa^+^ >150 mmol/L. Univariate analysis comparing parameters of eunatremic and hypernatremic athletes are presented in Tables 4, 5.

**Table 4:** Comparison of parameters of eunatremic and hypernatremic finishers.

	**Eunatremia**	**Hypernatremia**	***p* ^a^**
	**(*N* = 103)**	**(*N* = 25)**	
Age, years	38. [33–45]	38 [34–42]	**0.962**
Sex Male, *n* (%)	95 (92.2)	25 (100)	**0.15**
**Race**			**0.358**
VGI, *n* (%)	73 (70.9)	20 (21.)	
GTP, *n* (%)	30 (29.1)	5 (14)	
Weight (kg) pre-race	71.4 ± 8.5	75.1 ± 7.2	0.030^*^
Weight (kg) post-race	69 ± 8.2	71.7 ± 6.9	**0.099**
***p* ^b^**	<0.001^*^	<0.001^*^	
Weight loss (kg)	−2.3 ± 1.2	−3.4 ± 1.1	<0.001^*^
Weight loss (%)	3.3 ± 1.6	4.5 ± 1.3	<0.001^*^
BMI (kg/m^2^) pre-race	23.1 ± 1.7	23.3 ± 1.7	**0.577**
BMI (kg/m^2^) post-race	22.3 ± 1.7	22.2 ± 1.6	**0.81**
***p* ^b^**	<0.001^*^	<0.001^*^	
TBW (L) pre-race	46 [41.1–48.9]	47.2 [45.4–50]	**0.074**
TBW (L) post-race	45.2 [41–48.5]	46.6 [44.5–48.2]	**0.222**
***p* ^b^**	<0.001^*^	<0.001^*^	
Change in TBW (L)	−0.54 ± 1.3	−1.33 ± 1	0.001^*^
Change in TBW (%)	1.1 ± 2.7	2.7 ± 1.9	0.001^*^
Fluid intake (ml/h)	900 [700–1,100]	900 [750–1,150]	**0.848**
Fluid intake (ml/BMI/h)	39.5 [30.1–49.4]	38.9 [32.2–46.7]	**0.739**
**Whole blood parameters**			
Na^+^ (mmol/L) pre-race	139 [138–141]	140 [139–141]	**0.279**
Na^+^ (mmol/L) post-race	142 [140–144]	147 [146–149]	<0.001^*^
***p* ^b^**	<0.001^*^	<0.001^*^	
Change in Na^+^ (mmol/L)	2.5 ± 2.8	7.8 ± 2.3	<0.001^*^
K^+^ (mmol/L) pre-race	4.6 [4.2–5.1]	4.5 [4.1–4.9]	**0.414**
K^+^ (mmol/L) post-race	4.7 [4.3–5]	4.5 [4.2–5.1]	**0.575**
***p* ^b^**	**0.076**	**0.412**	
Glucose (mg/dL) pre-race	84 [78–90]	84 [74–88]	**0.743**
Glucose (mg/dL) post-race	103 [84–117]	104 [95–121]	**0.503**
***p* ^b^**	<0.001^*^	<0.001^*^	
EPOsm (mOsm/kg) pre-race	283 [281–286]	284 [281–287]	**0.342**
EPOsm (mOsm/kg) post-race	290 [286–294]	301 [298–303]	<0.001^*^
***p* ^b^**	<0.001^*^	<0.001^*^	

a *Comparison between Eunatremia and hypernatremia.*

b *Comparison between pre- and post-race.*

* *p < 0.05.*

*Quantitative parameters are expressed in mean (±standard deviation) or median [interquartile range].*

*VGI, Vitoria-Gasteiz Ironman; GTP, Grand Trial of Peñalara; BMI, body mass index; TBW, total body water; Na^+^, sodium; K^+^, potassium; EPOsm, effective plasma osmolality*.

**Table 5:** Additional parameters of athletes classified as eunatremic or hypernatremic at the end of their race.

	**Eunatremia**	**Hypernatremia**	***p* ^a^**
	**(*N* = 41)**	**(*N* = 12)**	
**Additional blood parameters**			
POsm (mOsm/kg) pre-race	303.6 ± 3.8	306 ± 4.8	**0.133**
POsm (mOsm/kg) post-race	313.8 ± 6	324.4 ± 3.2	<0.001^*^
***p* ^b^**	<0.001^*^	<0.001^*^	
Change in POsm (mOsm/kg)	10.2 ± 6.2	18.4 ± 5.8	<0.001^*^
Copeptin pre-race (pmol/L)^c^	**<15**	**<15**	**1**
Copeptin post-race (pmol/L)	89 [80.1–97.2]	85.6 [79.1–99.2]	**0.524**
***p* ^b^**	<0.001^*^	0.002^*^	
Change in Copeptin (pmol/L)	74 [65.1–82.2]	70.6 [64.1–84.2]	**0.524**
Pro-BNP (pg/ml) pre-race	20 [18–31.5]	20 [20–29.8]	**0.974**
Pro-BNP (pg/ml) post-race	241 [134–470.5]	125.5 [81–197.8]	0.026^*^
***p* ^b^**	<0.001^*^	0.002^*^	
Change in Pro-BNP (pg/ml)	201 [114–437.5]	105 [58.8–174.8]	0.025^*^
Urea (mg/dl) pre-race	41.5 ± 8	44.3 ± 8.7	**0.345**
Urea (mg/dl) post-race	62.4 ± 15.2	56.8 ± 6	**0.063**
***p* ^b^**	<0.001^*^	0.001^*^	
Creatinine (mg/dl) pre-race	0.9 [0.8–1]	0.9 [0.8–1]	**0.331**
Creatinine (mg/dl) post-race	1.1 [1.1–1.4]	1.2 [1.1–1.4]	**0.236**
***p* ^b^**	**<0.001**	**0.002**	
Leucocytes (x10^3^) pre-race	6 [5.1–7.3]	5.4 [5.2–6.3]	**0.438**
Leucocytes (x10^3^) post-race	14.2 [12.2–16.1]	15.7 [14.9–17.4]	**0.019**
***p* ^b^**	<0.001^*^	0.002^*^	
Hematocrit (%) pre-race	44.9 ± 3	46.1 ± 2.6	**0.207**
Hematocrit (%) post-race	43.9 ± 2.9	45.8 ± 2.7	**0.051**
***p* ^b^**	0.003^*^	**0.268**	
Hemoglobin (g/L) pre-race	14.8 [14.2–15.7]	15.5 [15–15.9]	**0.238**
Hemoglobin (g/L) post-race	14.9 [14.1–15.6]	15.3 [15.1–16]	**0.104**
***p* ^b^**	**0.286**	**0.344**	
**Urine post-race parameters**			
Osmolality (mOsm/kg)	901 [726–1049]	957 [869–1007]	**0.294**
Na^+^ (mmol/L)	56 [34–95]	107 [57–144]	**0.085**
K^+^ (mmol/L)	155 [129–206]	148 [125–276]	**0.79**

a *Comparison of parameters of athletes finishing in eunatremia vs. hypernatremia.*

b *Comparison of parameters pre- and post-race.*

c *Copeptin levels were indetectable in all finishers. For statistical analysis, a value of 15 pmol/L was used.*

* *p < 0.05.*

*Quantitative parameters are expressed in mean (± standard deviation) or median [interquartile range].*

*POsm, plasma osmolality; Na^+^, sodium; K^+^, potassium*.

No significant difference in mean salt intake was observed between athletes developing hypernatremia and those remaining eunatremic in GTP (5.350 vs. 9.730 g, *p* = 0.180) nor in VGI (4.510 vs. 5.240 g, *p* = 0.671). Salt intake was not correlated with BNa^+^ levels in either event.

The mean BNa^+^ increment was higher in those losing >3% of BW than in those who did not, both in GTP (3.65 ± 3.06 vs. 0.87 ± 3.1 mmol/L, *p* = 0.012) and VGIfin (4.94 ± 3.21 vs. 2.1 ± 3.1 mmol/L, *p* < 0.001). In both GTP and VGIfin, the change in BNa^+^ was negatively correlated with the change in BW (*r* = −0.486, *p* = 0.03; and *r* = −0.469, *p* < 0.001, respectively) and with the change in TBW (*r* = −0.235, *p* = 0.017; and *r* = −0.286, *p* = 0.005, respectively).

EPOsm increased over both races, in GTPfin: 2.2 ± 2.5% (range: −2.9–7.2), and VGIfin: 3.1 ± 2.5% (range: −3–8.9).

However, six (16.6%) GTPfin and 10 (10.6%) VGIfin experienced a fall in EPOsm. In athletes showing a EPOsm descent, BNa^+^ fell a mean of −2.7 ± 1 mmol/L (range:−4 to−1) in GTPfin, and −1.8 ± 1.2 mmol/L (range:−4 to 0) in VGIfin. BW also decreased a mean of −2.1 ± 1.7% (range: 0.1 to−4) in these GTPfin, and −1.8 ± 1.2% (range: 0 to−3.3) in these VGIfin. TBW decreased a mean of −0.5 ± 2.7% (range: 1.1 to−2.1) in GTP, but increased a mean of 0.5 ± 2.4% (range:−4.4 to 3.9) in VGIfin. Median FI in ml/h as well as in ml/BMI/h was not significantly different when comparing those showing a decrease in EPOsm with those who did not (931 vs. 933 ml/h, *p* = 0.596; and 39.7 vs. 40.6 ml/BMI/h, *p* = 0.789, respectively).

The median GTP finishing time was lower in participants developing hypernatremia than in those remaining eunatremic (17:34 h vs. 19:36 h, *p* = 0.018). In the VGI group, statistical differences between the finishing time medians of these subgroups were not observed (10:45 h vs. 10:46 h, *p* = 0.874). When the finishing time of those who lost >3% of BW was compared to those who did not, no differences were found in GTPfin (*p* = 0.314) nor VGIfin (*p* = 0.860).

### Additional Biochemical Parameters

In both GTP and VGI participants, copeptin was below detection level at baseline, ascending significatively post-race. No differences in copeptin levels between hypernatremic and eunatremic athletes were found. Participants showing a descent in BNa^+^ also had an elevation in copeptin levels. The change in copeptin levels did not correlate with BNa^+^, TBW, POsm, EPOsm, nor their changes from pre- to post-race.

Pro-BNP increased significantly during both races. However, the median Pro-BNP increment was lower in hypernatremic than eunatremic finishers of the entire group (125.5 vs. 241 pg/ml, *p* = 0.026). In VGIfin, a negative correlation was detected between Pro-BNP and copeptin concentrations (*r* = −0.400, *p* = 0.040), but not among GTPfin (*r* = 0.02, *p* = 0.910) nor in the entire group.

## Discussion

Our findings indicate that athletes hydrating according to 3IE-AHCD recommendations present a low incidence of hyponatremia, as compared with the findings of previous studies, without an increase in marked hypernatremia (1, 2, 6). Drinking according-to-thirst permits a FI tailored to the athlete's specific needs. This contrasts with recommendations for specific quantities of FI (“one-size-fits-all”) (5, 7).

Only 2 of the 126 (1.6%) athletes developed post-race hyponatremia (rate of 15 per 1,000). In both cases, hyponatremia was mild (134 mmol/L). These results contrast with the incidences ranging from 13 to 51% described in previous studies of similar sporting events (14, 16–25). The 15% hyponatremia rate at the Ironman European Championship event in Frankfurt 2017 is of particular note, given its similar characteristics to the VGI race (26). In the current study, drinking according-to-thirst, with specific placing of provisioning posts, almost entirely prevented development of hyponatremia in athletes completing their race.

The median FI of the athletes was 800 ml/h in GTPfin, and 900 ml/h in VGIfin, ranging from 400 to 1,500 ml/h and 500–1,500 ml/h, respectively. No athlete drank more than 1,500 ml/h, a risk factor for hyponatremia (27). However, athletes are often told to drink more than what thirst indicates. Many webpages encourage athletes to drink large amounts of liquid over short periods of time, with potentially fatal consequences (16, 19, 20, 28, 29). Hoffman et al. (21) reported the low quality of information on internet, even on webpages considered 'scientific'. In fact, only 7.3% of 110 webpages highlighted the need to drink according-to-thirst. Only 50% mentioned the risk of hyponatremia produced by excessive FI. Others recommend a FI of 400–800 ml/h during exercise (30). Yet, in the current study, the FI rate of 47% of GTPfin and 69 % of VGIfin was outside of this recommended range.

FI should be based on thirst, the physiological mechanism that determines the volume of liquid to be ingested. Thirst, together with AVP, exert a control of water homeostasis so precise (22) that EPOsm is normally maintained within strict limits (27). In fact, in the current study, no athlete had a post-race EPOsm ≥308 mOsm/kg. Non-thirst-based fluid recommendations are unphysiological, and do not take into account specific characteristics of athletes/races.

We observed a hypernatremia rate within the previously described range from 2 to 52% (31–34), with 14% in GTPfin and 21% in VGIfin. The development of hypernatremia did not appear to negatively affect athletic performance, as based on race-completion times. In fact, although no differences were observed between finishing times of hypernatremic and eunatremic athletes in VGI, the finishing time of GTP athletes developing hypernatremia was actually better than in those eunatremic. All athletes presented a BNa^+^ <150 mmol/L. Furthermore, no athlete was attended by the medical team for reasons other than musculoskeletal complaints. Thus, hypernatremia, when present, was mild, and not clinically apparent.

A majority of VGI (61%) and GTP (56%) finishers lost >3% of BW, but only one athlete presented >5% weight loss (5.2%). Although a BW loss >3% can be considered clinically significant, no athlete in this group presented a EPOsm >307 mOsm/kg, none collapsed, nor did any require medical attention upon race completion. Furthermore, as was the case for athletes with hypernatremia, there was no apparent negative impact on their athletic performance, as based on race-completion times.

Urinary sodium concentrations [UNa^+^] confirm that the hypernatremic runners were not hypovolemic. The median [UNa^+^] of 107 mmol/L, with no athlete presenting a level below 32 mmol/L, indicates a normal effective circulating volume. Had they been hypovolemic, [UNa^+^] would have been below 30 mmol/L.

The failure of athletes to replace 100% of BW losses from *ad libitum* FI has been described as “involuntary” or “voluntary” dehydration (35, 36). Laboratory and field data, however, suggest that the body primarily defends POsm, and not body water during prolonged endurance exercise (37–44). Furthermore, complete replacement of fluid loss is accompanied by a reduction in BNa^+^ during prolonged endurance exercise (45–48), without offering any performance benefit (48, 49). In the current study, a minority of athletes in both events showed a post-race EPOsm reduction, accompanied by a fall in BNa^+^. This occurred despite the majority of them presenting BW losses and having a FI similar to those not showing a EPOsm reduction. These findings elucidate the risk of hyponatremia behind an excessive FI. If the aforementioned athletes had consumed fluids with a goal of replacing 100% of BW loss, many more participants could have become hyponatremic.

Copeptin, a marker for AVP secretion (50–52), rose from undetectable levels pre-race in all participants to markedly high levels post-race. The low pre-race copeptin levels can be explained by the fact that all athletes drank without thirst during the 24 h prior to the race, thus inhibiting AVP/copeptin secretion (30). High post-race copeptin and urine osmolality levels were found even in athletes exhibiting a reduction in BNa^+^, ruling out excessive fluid intake as the driving force behind the sodium descent in these individuals. We found no correlation between POsm or EPOsm and post-race copeptin levels, nor between their changes, nor was copeptin correlated with BNa^+^ or TBW. These findings were to be expected, since the secretion of AVP, and thus copeptin, from the posterior pituitary is influenced by many non-osmotic stimuli that occur during exercise, including nausea, pain, stress, cytokines, and physical activity itself.

In agreement with previous reports (53, 54). Pro-BNP increased significantly over both races. There was a negative correlation between copeptin and Pro-BNP levels in VGIfin, as has been also previously described. In fact, Hew-Butler et al. proposed that Pro-BNP participates as a counter-regulator of AVP secretion when the latter is stimulated by hypovolemia (55). Tachycardia-induced acute myocardial stress could also elevate Pro-BNP levels (56, 57). Harris et al. (58) have suggested that pro-BNP could be involved in the development of endurance-sport hyponatremia. However, in the current study, changes in pro-BNP were not correlated with changes in BNa+, nor post-race BNa+ levels.

Our study has several important limitations. First of all, few finishers were women.

Secondly, we did not compare the study group to the athletes in the same races who finished their race, yet did not participate in the study. Instead, we have seen a low rate of hyponatremia as compared to the findings reported in previous studies.

Thirdly, athletes that did not complete their race were not evaluated, and therefore we cannot rule out dysnatremia in non-finishers. However, the fact that not a single athlete, regardless of whether they finished the race or not, was attended by the medical team for anything other than musculoskeletal complaints seems to rule out at least moderate or severe dysnatremia. Furthermore, only finishers have been studied in previous reports, thus permitting us to compare our results with those presented in earlier publications.

Fourthly, fluid intake was self-reported, *via* questionnaire, and could be imprecise.

In conclusion, our study validates the 3IE-AHCD recommendations for FI based on thirst during ultra-endurance events. The incidence of hyponatremia, a potentially fatal condition, was low as compared with the findings of previous studies, and hyponatremia mild, without induction of clinically significant hypernatremia, nor an apparent negative repercussion on athletes, as based on race times. Coaches/trainers, athletes and event personnel/organizers should be made aware that athletes should only drink the quantity of liquid that their thirst demands. Educational sessions on hyponatremia should be held, assuring recognition of athletes needing urgent attention.

## Data Availability Statement

The raw data supporting the conclusions of this article will be made available by the authors, without undue reservation.

## Ethics Statement

The studies involving human participants were reviewed and approved by Ethics and Clinical Research Committee of the HCSC (internal code 17/018-E). The patients/participants provided their written informed consent to participate in this study.

## Author Contributions

DL, IR, and JV: conceptualization. DL, MC, and GS: methodology. DL, JR-S, MC, GS, MR, AC-P, IR, and JV: validation, writing—review and editing, supervision, and reviewing. DL, MC, and JR-S: formal analysis. DL, MC, JR-S, and IR: investigation. DL, JR-S, IR, and JV: writing—original draft preparation. All authors have read and agreed to the published version of the manuscript. All authors contributed to the article and approved the submitted version.

## Conflict of Interest

The authors declare that the research was conducted in the absence of any commercial or financial relationships that could be construed as a potential conflict of interest.

## Publisher's Note

All claims expressed in this article are solely those of the authors and do not necessarily represent those of their affiliated organizations, or those of the publisher, the editors and the reviewers. Any product that may be evaluated in this article, or claim that may be made by its manufacturer, is not guaranteed or endorsed by the publisher.
