# Supplementary material for: Exercise-Induced Hyponatremia: An Assessment of the International Hydration Recommendations Followed During the Gran Trail De Peñalara and Vitoria-Gasteiz Ironman Competitions
Source: Front Nutr. 2022 Feb 21;8:781229. doi: 10.3389/fnut.2021.781229 (PMC8898836; doi:10.3389/fnut.2021.781229)
Supplement: Supplementary file 1 [file Data_Sheet_1.DOCX]

**RECOMMENDATIONS PROVIDED TO COMPETING ATHLETES**

Hydration guidelines based on scientific evidence must be made known to athletes.

1. **Fluid intake higher than that indicated by thirst has not been shown to reduce exercise-related fatigue, the frequency of muscle cramps, nor acute coronary events** (1).

2.- **A small degree of dehydration is easily tolerated, with minimum risk to athletes’ health** (5).

3.- Strategies directed towards preventing both dehydration and overhydration during exercise are recommended.

a) **Drink as a function of thirst.** The loss of liquid through sweat and urine are extremely variable from one athlete to the next, are dynamic, and influenced by multiple factors. Therefore, fixed hydration strategies are inadequate. The best way to individualize the hydration strategy for an individual athlete is to drink according to thirst, as a real-time, safe, effective hydration strategy (1,6).

**b) Reduce the number of posts offering liquids during the race.** This measure has been shown to reduce the incidence of hyponatremia. In Ironman triathlons, the recommendation is to place provision stations every 2.5 km, and every 20 km during the bicycle portion of the race, as is done at the Triathlon of Vitora-Gasteiz (7). In any case, more studies are needed to identify the best strategy regarding the number and position of provision posts, as a function of the weather and other environmental variables.

c) **Monitor weight change induced by the race.**  Monitoring body weight provides a reasonable, albeit imprecise indication of hydration status. Athletes should NOT gain weight during a long-distance competition. Weight gain indicates an excessive fluid intake, and implies a degree of overhydration (1). Therefore, athletes who gain weight during these types of events should reduce their fluid intake in future competitions.

d) **The USA Track and Field guidelines recommend a sweat test to determine the rate of sweating/hour under specific conditions**. However, the results of this test may. not be helpful, since ADH may not be suppressed during the race (1).

e) **Finally, the** **American College of Sports Medicine (ACSM) recommends the intake of 500-700 mg of sodium (22-30 mEq/L) for every liter of water, to make up for sodium losses in sweat, in these events** (8). In any case, this degree of sodium intake, together with the sodium provided in sports drinks, attenuates but does not protect from the risk of developing exercise-related hyponatremia that has been induced when overhydration has occurred.

Educational programs should be promoted to aid in recognition of the signs and symptoms of hyponatremia, permitting urgent medical assistance to athletes with suspected hyponatremia. The application of the forementioned measures reduces the risk for ultradistance event-induced hyponatremia, without reducing the athlete’s performance.

We hope you will enjoy the event, with energy and good health.

Good luck, ahtletes!

BIBLIOGRAFIA

1.-Hew-Butler T, Rosner MH, Fowkes-Godek S, Dugas JP, Hoffman MD, Lewis DP, et

al. Statement of the Third International Exercise-Associated Hyponatremia Consensus Development Conference, Carlsbad, California, 2015. Clin J Sport Med (2015) 25(4):303–20. 2.- Cruel J. 12 Easy Ways to Drink More Water Every Day. Self. (2015). Available from: http://www.self.com/story/how-to-drink-more-water

3.- Associated Press. Woman Dies after Water Drinking Contest. NBC news. com. (2007). Available from: http://www.nbcnews.com/id/16614865/ns/ us_news- life/t/woman-dies-after-water-drinking-contest/

4.- Hoffman MD, Bross TL III, Hamilton RT. Are we being drowned by over hydration advine on the Internet? Phys Sports med (2016) 44(4):343–8

5.- Sawka MN, Burke LM, Eichner ER, Maughan RJ, Montain SJ, Stachenfeld NS. American College of Sports Medicine position stand. Exercise and fluid replacement. Med Sci Sports Exerc (2007) 39(2):377–90.

6.- Sharwood K, Collins M, Goedecke J, Wilson G, Noakes T. Weight changes, sodium levels, and performance in the South African Ironman Triathlon. Clin J Sport Med (2002) 12(6):391 9

7.- Speedy DB, Rogers IR, Noakes TD, et al. Diagnosis and prevention of hyponatremia at an ultradistance triathlon. Clin J Sport Med 2000; 10:52

8.- Rosner MH, Kirven J. Exercise-associated hyponatremia. Clin J Am Soc Nephrol 2007; 2:151
